# Method Presentation of a New Integrated Orthodontic-Conservative Approach for Minimally Invasive Full Mouth Rehabilitation: Speed Up Therapy

**DOI:** 10.3390/biomedicines10102536

**Published:** 2022-10-11

**Authors:** Davide Foschi, Andrea Abate, Cinzia Maspero, Luca Solimei, Claudio Lanteri, Valentina Lanteri

**Affiliations:** 1 Private Practice, 40100 Bologna, Italy; 2 Department of Biomedical Surgical and Dental Sciences, University of Milan, 20142 Milan, Italy; 3 Fondazione IRCCS Cà Granda, Ospedale Maggiore Policlinico, 20142 Milan, Italy; 4 Department of Surgical Science and Integrated Diagnostics (DISC), University of Genova, 16126 Genova, Italy; 5 Private Practice, Casale Monferrato, 15033 Alessandria, Italy

**Keywords:** speed-up therapy, set-up, mock-up, clear aligners, smile aesthetics, orthodontics, full mouth rehabilitation

## Abstract

The materials available today allow for extensive oral rehabilitations in a non-invasive way, and often an orthodontic preparation is useful and, thanks to the use of clear aligners, is predictable and comfortable. A preliminary study of the wax-up, mock-up, and set-up allow the clinician to plan every aspect of the treatment in detail. Furthermore, the procedure offers the patient an intuitive and understandable view of the expected final result. The new proposed method, called “speed up therapy”, allows for the integration of the orthodontic set-up with the mock-up technique, simulating the occlusal and aesthetic components of the planned restoration, in all details. The clinical case presented, demonstrates step by step the predictability and clinical reliability of the proposed procedure. The final clinical result coincides exactly with the initial mock-up and demonstrates that the proposed method is predictable and reliable. The correct execution of the technique is rigorously customized, and its success is operator dependent, both for the clinical aspects and for the dental laboratory. Thus, the visualization of the objectives of the treatment constitutes a decisive support for the clinician and provides the patient with the possibility of benefiting from an immediate improvement by making it easier for them to accept a treatment plan. The visualization also includes an orthodontic phase that potentially lengthens the treatment but makes the realization more conservative and predictable.

## 1. Introduction

Dentists are often called upon to care for adult patients who, not conscious of the complexity of their problems, wish to improve their appearance. The causes of smile imperfections are manifold and are associated with each other [1]. Some of these causes are, for example, the evolution of untreated malocclusions at a suitable age, tooth loss not replaced by prostheses, tooth movement secondary to loss of periodontal support, occlusal changes due to inadequate conservative or prosthetic therapies, and tooth wear due to erosion and parafunctions. Dental erosion is a constantly increasing phenomenon, related to both environmental factors (mainly dietary and/or volitional) and organic factors of intrinsic origin (gastro-esophageal reflux) [2,3,4,5]. Due to the overlapping of hard tissue lesions of different etiology and the lack of homogeneous and universally accepted classification criteria, it is often difficult to diagnose [6,7]. Instead, its destructive potential is evident, in the most severe cases it can cause vertical dimension collapse, resulting in the occurrence of aesthetic impairment and functional alterations that require multidisciplinary interventions [8]. The traditional therapeutic approach involves the preparation of damaged dental elements and their prosthetic recovery, sacrificing parts of intact dental tissue and resorting not infrequently to endodontic therapy.

Thanks to adhesive techniques, vertical dimension and the correct occlusal morphology can be restored by minimally invasive techniques (direct or indirect additive approaches) that allow for a significant savings in biological tissue [8]. In many cases, preliminary orthodontic treatment can help to resolve various types of dental incongruities and correct irregularities in arch shape and occlusal plane or loss of vertical dimension, reducing the need for compromise solutions that could have negative repercussions on prosthetic work prognosis and customer satisfaction [9,10].

In orthodontics, it can be considered “adult” when the patient has completed the permutation of teeth and, above all, their skeletal growth potential, in relation to both sex and individual variables. Tissue modifications that occur at the end of growth and slow the biological response to orthodontic forces are characterized by the numerical reduction of connective cells in the ligament and alveolar bone, which acquires a more compact appearance due to the reduction of medullary spaces and vascular network [11,12]. The end of growth coincides with the inactivation and progressive synostosis of the sutures [13,14]; thus, the orthodontic approach of the adult can only contemplate dento-alveolar problems, without being able to address skeletal discrepancies; in cases of severity, these must be corrected surgically [15].

The choice of therapeutic devices is largely conditioned by the treatment goals and the requirements of performing complex root movements. The ideal ones should accomplish the required movements in a short time and with the best possible aesthetics. They should also have a simple structure to ensure the best comfort and predict the absence of pain to allow for easy management of orthodontic forces as well as accurate hygiene [16]. To perform orthodontic treatment, the cooperation of the adult patient is important: First, they must accept the equipment used, which can lead to aesthetic and social problems. In addition, they must have acquired complete control of the bacterial plaque and gingival inflammation before starting the treatment by carefully observing hygiene rules [17].

In response to these multiple needs, the usual choice is clear aligners that, thanks to today’s customized technologies, allow for a high three-dimensional control of the teeth that is adequate for the treatment of most cases [18,19,20]. In the presence of an important cross-sectional deficit of the palate, dental alignment will normally be preceded by a dental-alveolar expansion, by devices producing light and constant force [21,22].

From the point of view of the prosthetic treatment plan, the preliminary orthodontic preparation can reduce the spacing present in the arch and/or convey them in a single district (preferably in the posterior areas of the arch) in order to reduce the size of the restorations, optimize the shape of the coronal reconstruction, and allow a more conservative approach, with reduction of the subtractive preparation and therefore saving the biological heritage of the patient. In other words, the correct positioning of the teeth can facilitate the morphology of the conservative-prosthetic restoration and reduce the invasiveness of the preparation, while respecting the biological heritage of the patient [23,24,25].

The aim of this report was to present a new orthodontic-conservative integrated method for the minimally invasive rehabilitation of the full mouth, considering the most recent method reported in the scientific literature, and in terms of etiology, diagnosis, and treatment of lesions of erosive origin.

## 2. Case Report

**Figure 1:**
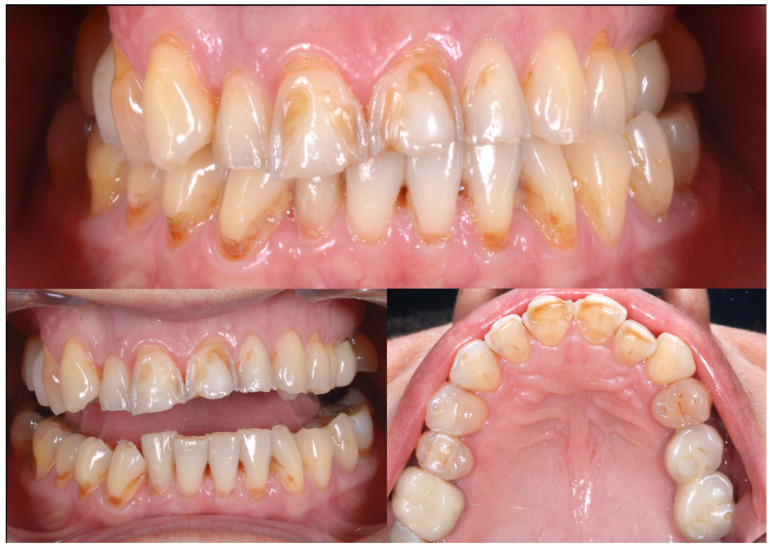
Intra-oral photos showing the extensive erosion of the frontal teeth.

**Figure 2:**
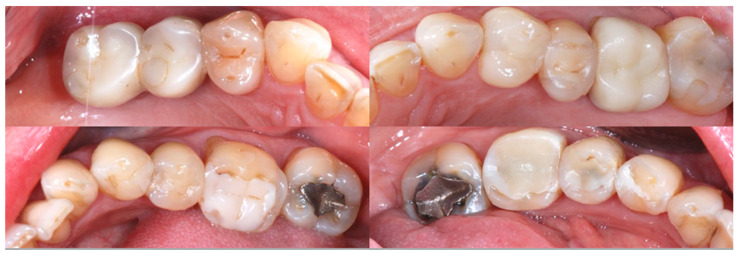
Incongruous conservative and prosthetic treatments visible at the occlusal surface of the posterior sectors.

**Figure 3:**
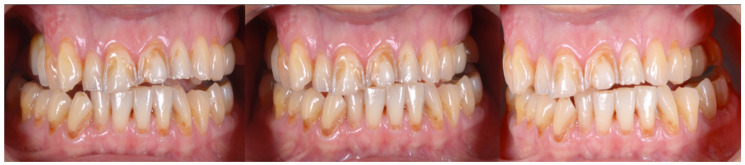
Occlusal changes that occurred as a result of the extensive tooth erosions compromised the physiological occlusal dynamics in protrusion and lateral movements.

A 54-year-old man came to the dental clinic reporting functional and aesthetic complications in relation to the numerous erosive lesions present in the upper and lower arch (Figure 1). At the clinical examination, the patient’s dentition showed extensive erosions at the palatal surfaces of the frontal elements and wear on the occlusal surface of the posterior sectors, resulting in loss of vertical dimension and tilting of the occlusal plane to the right. In addition, posterior cross bite and misalignment of the anterior sector were present in the upper arch, while in the lower arch, there was anterior crowding. Moreover, incongruous conservative and prosthetic treatments were visible at the occlusal surface of the posterior sectors (Figure 2). It was also observed that occlusal changes that occurred because of the extensive tooth erosions compromised the physiological occlusal dynamics in protrusion and lateral movements (Figure 3).

**Figure 4:**
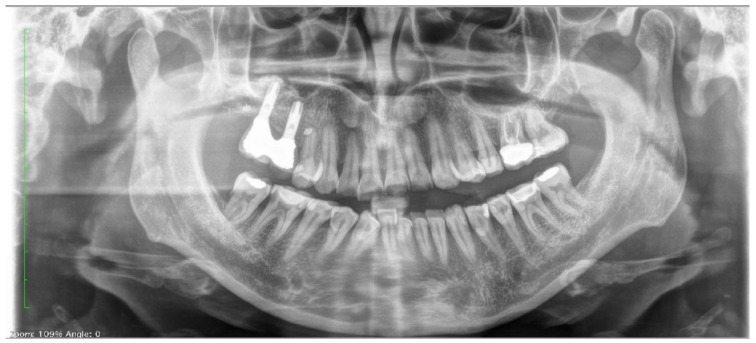
Pretreatment panoramic radiograph.

**Figure 5:**
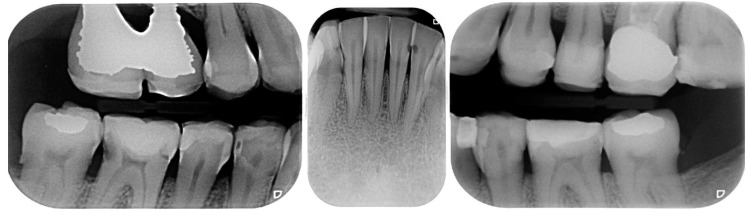
Pre-treatment evaluations by endoral radiographs.

Clinical and radiographic examinations revealed a good status of the periodontal tissue (Figure 4 and Figure 5).

### 2.1. Therapeutic Plan

In complex cases requiring an initial phase of pre-prosthetic orthodontics, it is useful to perform a set-up that, by simulating the outcome of orthodontic therapy, allows the dentist and orthodontist to plan together the final position of individual teeth and the distribution of spaces in the arches. Three-dimensional visualization of the result makes the treatment goals immediately understandable even to the patient, who can thus clearly understand the expected result and thus become more aware and cooperative. The shortcoming of the conventional orthodontic set-up is that it only visualizes the final position of the teeth without reference to other aspects that are fundamental to esthetic recovery, especially shape and color.

Our contribution to solving this problem is drawn from the already well-known “three-step technique” of Vailati et al. [26,27,28], but differs from it in materials and operative protocols. The most significant feature consists in integrating the diagnostic wax-up (wax-up) and mock-up with the orthodontic set-up, thus offering an anticipation of the result, complete with all details, through the close coordination of the dental team with the dental laboratory.

Step I: the diagnostic wax-up or wax-up: The first step is diagnostic wax-up, performed on the patient’s models for preliminary visualization of the final result regarding tooth position, crown morphology, and occlusion characteristics. Treatment simulation facilitates interdisciplinary communication and planning the timing of the different treatments required [27].

Step II: the mock-up: In the second step, on the basis of the wax-up, the composite mock-up is created directly in the mouth, which also allows for the aesthetic and functional components of the planned restoration to be accurately simulated, on the basis of individual anatomical and functional parameters [29,30]. According to the literature, if the vertical dimension loss is less than 0.5 mm, it is possible to intervene with direct restorations to protect the exposed dentin; if the loss is less than 2 mm, the choice will fall on direct composite restoration of both the occlusal surface of posterior elements and the palatal and vestibular surface of anterior elements; for defects greater than 2 mm, the prevailing indication suggests indirect restorations; finally, if the loss is more than 4 mm, the classical choice fell on conventional fixed prosthesis, but today it can also be achieved through the use of partial restorations [31].

The practitioner’s sensitivity will guide them in transferring the patient’s expectations into solutions of immediate understanding. The expectations of the dentist must also find a place in this phase, and the dentist will be able to make the patient aware of the details of the morphological customization. This phase ends when the functional and aesthetic feedback of the provisional solution reaches the full satisfaction of both parties.

Step III: the set-up: In the third step, the orthodontic set-up is performed. It aims to realize the mock-up that has already been evaluated and approved, changing the position of the teeth from a pre-prosthetic and not abstractly orthodontic perspective. In other words, in speed up therapy, the purpose of the set-up is to bring the natural teeth into the most favorable positions to minimize the final prosthetic preparation required by the realization of the mock-up. For example, in II/II malocclusion with proclined lateral incisors and retroinclined central incisors, an alignment with veneers would require very aggressive preparations that can be significantly reduced by preliminary orthodontic treatment of the mispositioned and abraded teeth. The mock-up represents the goal of the treatment, while the orthodontic set-up aims to bring the teeth (underlying the mock-up) into the optimal position for the purposes of ensuring the best biological sparing of the tissues that are to be reconstructed by prosthetic intervention and will therefore have to take on peculiar characteristics by design and clinical implementation. In essence, the orthodontic laboratory performs a series of aligners, of which one will impart a small movement to the affected teeth, programmed in consultation with the orthodontist. Individual teeth’s movements are broken down into fractions of 0.20 mm, and then aligners will be produced in the number necessary to achieve the programmed result [18]. The design of each aligner will be developed as if working on natural dentition, but the clinician will have to provide for the retouches of the mock-up-coated surfaces that will be necessary progressively, because of the change in position/relation of the teeth caused by the programmed orthodontic movements. During the creation of set-up, therefore, the vestibular aspect of the mock-up is considered as the final reference point for orthodontic correction.

Step IV: the orthodontic treatment: The special feature is that, during orthodontic movement, the initial provisional alignment obtained by mock-up will tend to become incongruous. It follows that any change of aligner will have to be accompanied by a modification of the mock-up equal to the planned movement. Perfecting the position of the natural tooth will ensure that the need for reconstruction/preparation is finally minimized and made perfectly predictable. A special aspect of the orthodontic phase, which is highly appreciated by patients, is that the teeth are moved without removing the mock-up, thus maintaining the esthetic benefit gained already at the beginning of treatment.

Step V: prosthetic finalization: At the end of orthodontic treatment, the alignment of the teeth will be optimal while the morphology of the mock-up will be significantly reworked, because of the retouching required to accommodate tooth movement. With the introduction of new materials available for modern conservative dentistry, it is possible to rehabilitate with direct or indirect technique elements affected by blemishes, discoloration, and wear with a satisfactory functional and esthetic result [8,9,26]. At this point, the clinician proceeds with the removal of the residual mock-up and performs the definitive reconstruction, choosing the technique that they prefer and taking care to reproduce the clinical, functional, and esthetic outcome, which is liked by the patient, even before the start of therapy.

Thus, the steps involved in our proposed technique follow the following order: diagnostic wax-up, mock-up, orthodontic set-up based on the mock-up, orthodontic treatment with clear aligners (without removing the mockup), and prosthetic finalization (possibly preceded by sectional temporaries).

### 2.2. Clinical Example

**Figure 6:**
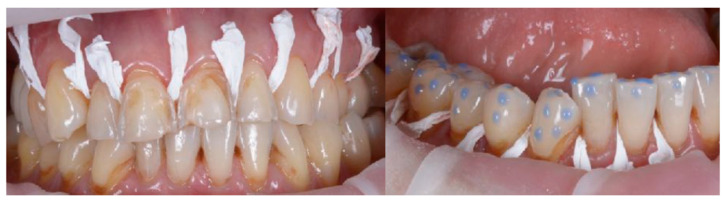
Selective etching of surfaces and application of adhesive system for better mock-up stability.

**Figure 7:**
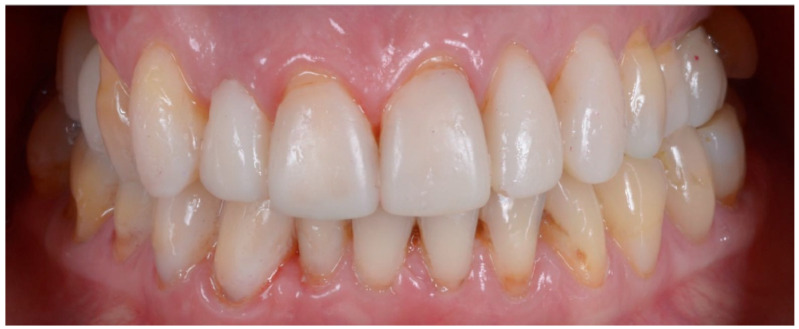
Initial mock-up made without tooth preparation (additive approach) through the use of a self-curing composite resin.

To describe the details of the proposed method, regarding the treatment of the frontal sectors of both arches, we report below a demonstration clinical case. The treatment plan proposed in this clinical case perfectly mirrors the procedure proposed in the previous paragraph (Steps I-II): treatment planning by wax-up, mock-up, and set-up: the performance of wax-up and mock-up allows for the visualization of the final result and three-dimensional assessment of the orthodontic movements required for the purpose of optimal use of the available dental structures, minimizing the invasiveness of the final restoration (Figure 6 and Figure 7).

**Figure 8:**
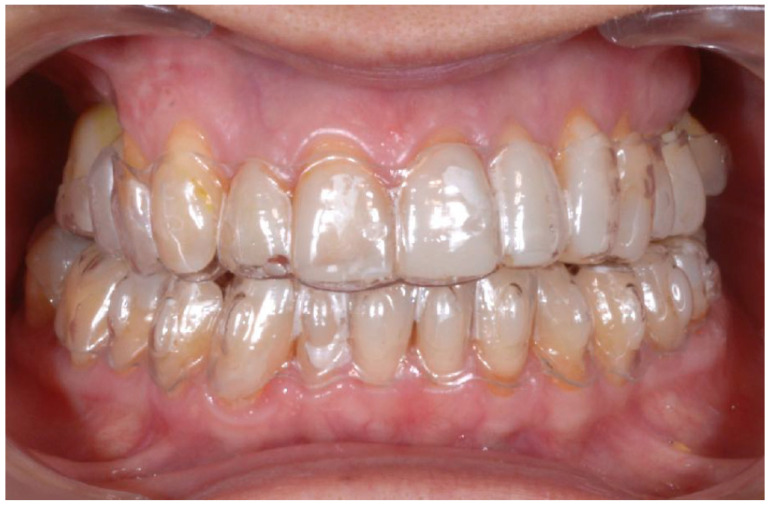
Orthodontic treatment with customized aligners based on the mock-up.

**Figure 9:**
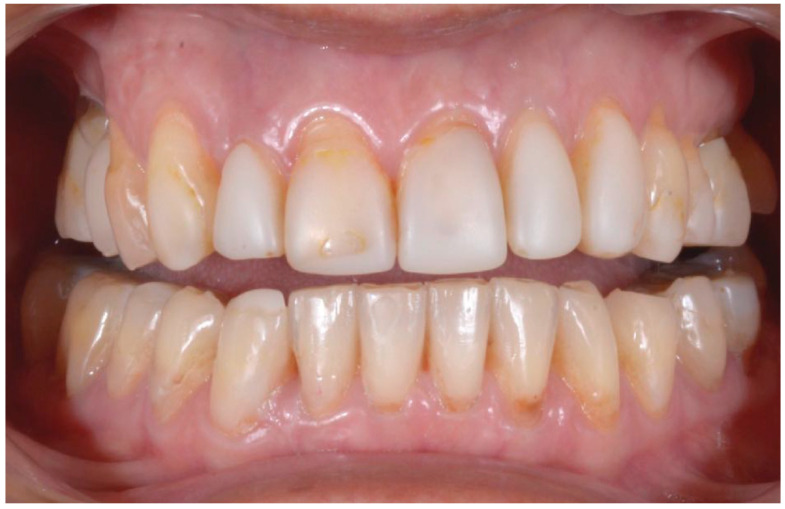
Result after 6 months of aligners.

Set-up and orthodontics (Steps III-IV): The orthodontic step is the next step after mock-up creation and the creation of a clinical validity of vertical dimension elevation. Each new aligner will exert different pressure on individual teeth, programmed with the digital set-up. It will be the clinician’s job to perform stripping and occlusal retouching of the mock-up to allow the teeth to move in the predetermined direction and amount (Figure 8). After 6 months of aligners, the goals of orthodontic treatment have been achieved, and the case is ready for finalization (Figure 9).

**Figure 10:**
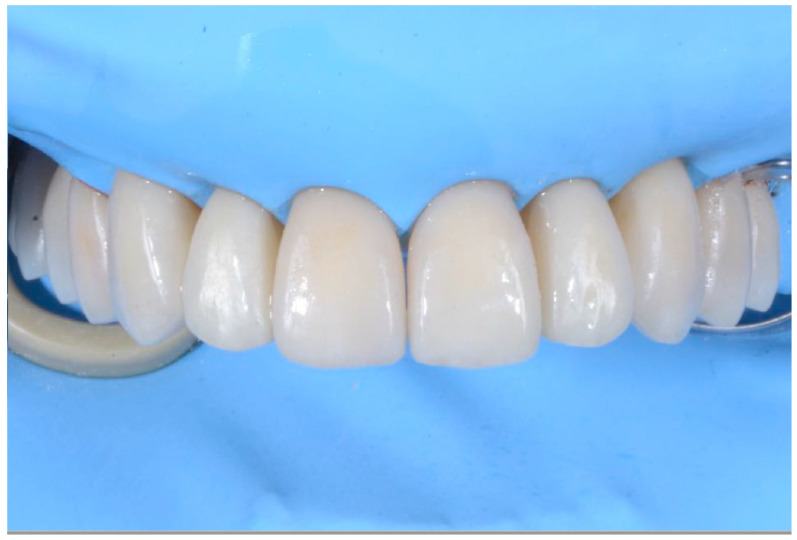
Adhesive cementation of prosthetic artifacts (overlays, onlays, and veneers) using an adhesive technique, dry field.

Prosthetic finalization (Step V): This phase involves the aesthetic and functional reconstruction of the arches through the placement of definitive restorations by employing adhesive techniques. The choice of materials for reconstruction is a point of fundamental importance, in order to be evaluated according to the needs of the individual patient, favoring an attitude as conservative as possible. If the technique has been correctly performed in each step, the exact correspondence with the functionalized mock-up will be verified (Figure 10).

### 2.3. Outcome Achieved

**Figure 11:**
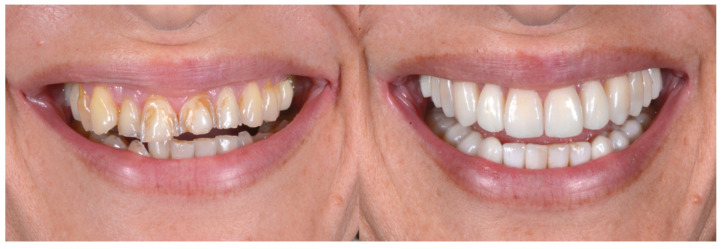
Comparison of the initial and final situation.

With the aim of objectively evaluating the achieved results, we used as a reference a checklist that reports the anatomo-functional and aesthetic conditions that most frequently induce the adult patient to undergo more or less complex rehabilitative treatment (Table 1 and Table 2). The correct execution of the orthodontic-conservative procedure allowed us to achieve, with excellent approximation, the planned result, both from the functional point of view and from the point of view of smile aesthetics (Figure 9, Figure 10 and Figure 11).

**Figure 12:**
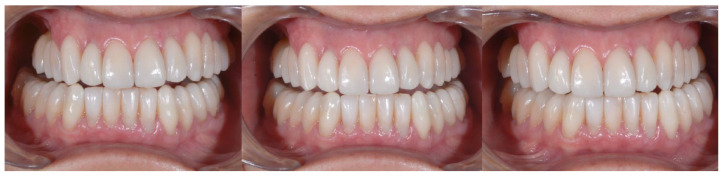
Physiological occlusal dynamics in protrusion and lateral movements.

**Figure 13:**
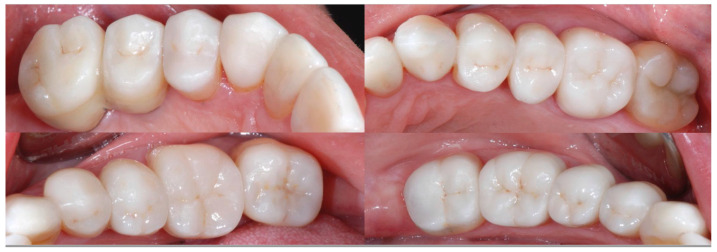
Details of posterior quadrant reconstruction with recovery of the vertical dimension, chewing efficiency, and aesthetics.

Orthodontic treatment and anatomical reconstruction of eroded teeth allowed us to create the reconstitution of physiological occlusal dynamics (Figure 12 and Figure 13).

**Figure 14:**
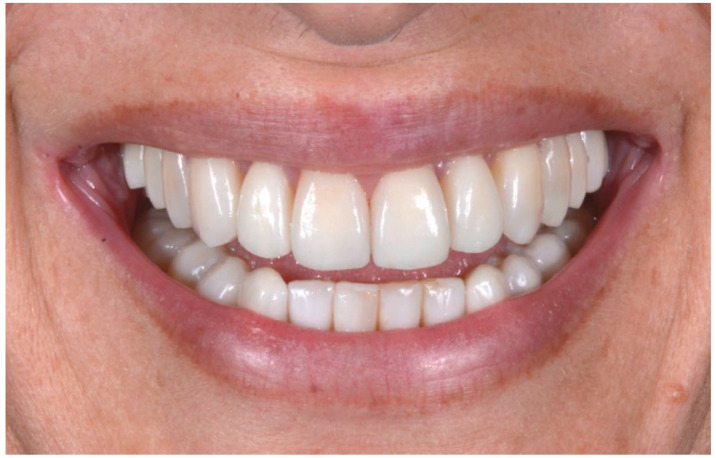
Details of posterior quadrant reconstruction with recovery of the vertical dimension, chewing efficiency, and aesthetics.

**Figure 15:**
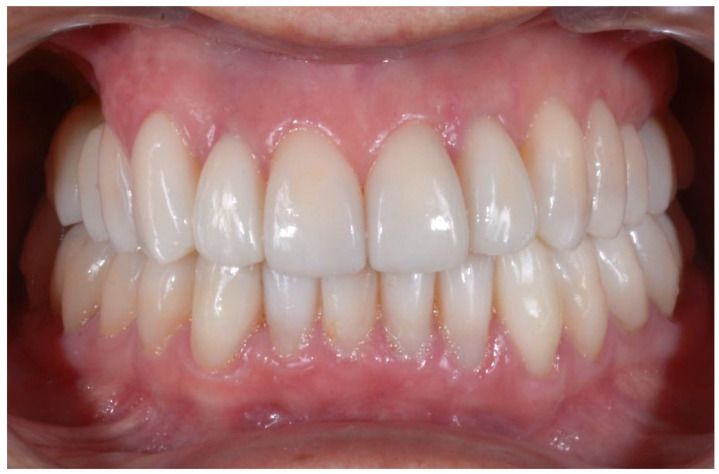
Clinical follow-up at 24 months.

**Figure 16:**
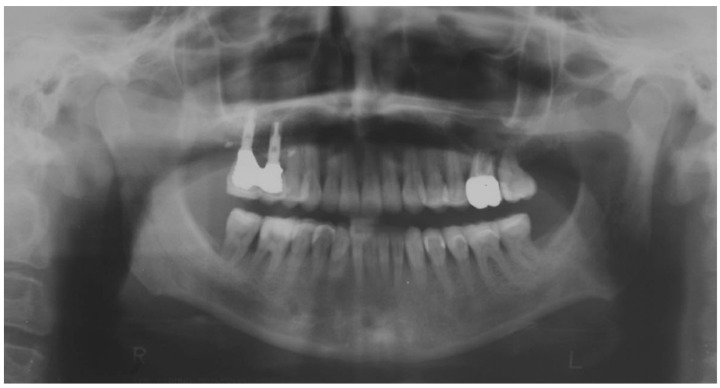
Orthopantomography at 24 months.

The correct execution of the orthodontic-conservative procedure allowed us to achieve, with excellent approximation, all the planned goals (Figure 14). Stability of outcome was confirmed by a clinical and radiographical follow-up at 2 years after the finish of therapy (Figure 15 and Figure 16).

## 3. Discussion

Several authors have proposed different criteria to define the aesthetic canons of a harmonious smile [1,29]. The ideal smile is an individual datum, generated by the integration of numerous components capable of stimulating a wide range of emotional reactions. Our case provides us the opportunity to summarize the most well-known ones, in order to be able to identify whether the correct dento-labial ratios have been achieved. We therefore obtained some of the best-known esthetic parameters from the literature and then subjected our results to verification (see Table 2—Aesthetic parameters of the smile). The presented clinical case, considering the parameters listed in Table 1, demonstrated that the planned goals were achieved. In particular, the final clinical image coincides with the initial mock-up, demonstrating that the proposed method proved to be predictable and reliable [30,32].

One of the greatest advantages in such particular case is that the “eroded” teeth with exposed dentin do not suffer any more sensitivity and possible worse degenerations due to the application of the segmented full mock-up at the beginning of the treatment [4,33,34]. Every mock-up/set-up modification was tested and functionalized directly in the mouth using the same reversible approach: sandblast of the existence surfaces, bonding application, and self-curing/light curing resin application without the need to anesthetize the patient, which were very helpful in regulating the occlusal contacts differently from what occurs in a classical prosthodontics approach. From the patient’s point of view, the most appreciated aspects were the aesthetics and the comfort during the provisional phase, so we could say that the immediate aesthetic improvement and the possibility to be additive and not subtractive were the real benefits given by this combination of treatments. The choice of the transparent aligners was the perfect integration of the orthodontic treatment, given the same concept: additive appliances, soft forces, preview of the final result, and more comfort for the patient. Moreover, the choice to use clear aligners stems from the fact that they do not significantly affect the periodontal and microbiological conditions of the oral cavity. The literature reported that the use of clear aligners should be considered as a valuable therapeutic option that has no significant impact on oral and microbiological parameters if compared with untreated patients [35,36,37].

The proper execution of the technique is rigorously customized, and its success is certainly operator dependent, both for the clinical aspects and for the dental laboratory. The present case report allows us to emphasize the importance of the treatment plan, which must be planned in detail for procedures and timing, being aimed at achieving therapeutic objectives in the shortest possible time. It is also very important that the orthodontic phase involves the use of simple appliances to minimize patient discomfort and difficulties in maintaining oral hygiene, which must always be kept impeccable. The particularity of this type of patient, characterized by great variability in individual clinical photos, makes it effectively impossible to have homogeneous samples on which to base statistically significant studies for the purposes of evidence-based medicine. Rather, the evidence of reference will be based on clinical observations and customer satisfaction.

Our patients are increasingly exigent about medical and dental care in general, with particular regard to treatments with significant esthetic implications. Preliminary visualization of treatment goals, detailed and immediately understandable, introduces an extraordinary new communication instrument that brings significant added value into our relationships with our patients. From a marketing point of view, such a concrete visualization of the planned result facilitates acceptance by patients, who show that they greatly appreciate the realistic and rapid response to their aesthetic requests. Moreover, of note is the full respect for individual biological characteristics and the preventive aspects towards possible iatrogenic damage inherent in the technique [38]. The additive approach is today the first option to be considered when treating serious problems of dental wear. The adhesive prosthesis characterized using partial restorations, such as onlays, overlays, and veneers, is now preferred and sought not only by professionals, but also by patients. Exploiting the variation of the vertical dimension of the occlusion allows for operations to occur whilst preserving the residual dental structures to the maximum and reducing the subtractive procedures to a minimum. Whatever gnathological philosophy the clinician may prefer, the idea “to test” the project in a totally reversible way, using complete mockups, represents a reliable, dynamic, cost-effective, and comfortable method for patients.

## 4. Conclusions

In conclusion, the correct execution of the present orthodontic-conservative approach allowed us to obtain, with excellent approximation, the planned results, both from the aesthetics and functional point of view. The collaboration between dental office and laboratory plays a key role in the planning and achievement of the rehabilitation process. The comparison between the needs of the clinician and those of the dental technician allows for a rationalization for situations where it is preferable to segment the realization of final restorations, rather than proceed with the simultaneous realization of complete arches.

The development of new hybrid materials and digital workflow procedures allows us today to make completely adhesive rehabilitations, pushing the limit of the additive approach beyond what seemed impossible only few years ago. The challenge for the coming years will be to obtain therapeutic results that can come as close as possible to the patient’s expectations.

## Figures and Tables

**Table 1:** Summary objective examination of the patient before and after treatment.

	Before	After
Disharmonious smile	Yes	No
Dyschromia, dysmorphia, abrasions, and erosions of teeth	Yes	No
Anterior crowding in the upper arch	Yes	No
Anterior crowding in the lower arch	Yes	No
Upper transverse discrepancy	Yes	No
Presence of buccal corridors or black tunnels	Yes	No
Curve of Spee alteration	Yes	No
Curve of Wilson alteration	Yes	No
Functional movements with altered guides	Yes	No
Pain and/or noise ATM	Yes	No

**Table 2:** Smile aesthetic parameters.

Parameter Examined	Clinical Significance	Result Obtained
Smile symmetry	Smile symmetry refers to the mirror agreement between the two sides of the mouth but can also consider the bi-pupillary line, the incisal line, and the labial commissure.	YES
Horizontal symmetry	It originates from the presence of similar elements placed in a regular sequence, as verified in a dentition with teeth well aligned in the horizontal plane.	YES
Vertical symmetry	Recall the same principle as above, referring to the vertical direction.	YES
Repeated report	It indicates the division of space into portions that may not be identical in shape and size but arranged to generate a harmonious connection between them. This is what is verified between opposing hemiarches with well-arranged teeth, inspiring a sense of order and balance.	YES
Prospective effect	The contour of the buccal surface and the alignment of the inclined planes of the teeth is decisive in generating a correct perspective effect. The different length or strong color difference of even one element can impair the perspective effect and compromise the sense of harmony of the whole.	YES
Lip height	Useful distinction in static and dynamic harmony. The average height of the upper vermilion is 7.1 mm in the male and 7.7 mm in the female. The lower vermilion is normally more extensive, about 10 mm on average; these are statistical values, with wide individual variations.	YES
Lip line	The height of the upper lip, relative to the upper central incisors, can be classified as low, medium, or high, on the basis of the amount of crown exposure.	YES
Smile line	Smile line is a curved line passing through the incisal margin of the upper incisors, parallel to the inner margin of the lower lip.	YES
Curvature of the upper lip	With superior convexity, it extends from the center toward the lateral triangular spaces. When rectilinear or worse with inverted convexity, it gives the subject a sad and unattractive expression.	YES
Frontal axial alignment	The smooth slope of the long axis of the front elements helps generate a sense of regularity.	YES
Tooth alignment in the arch	Recalls the anatomical harmony represented by the correct positioning of teeth in the center of the alveolar ridge.	YES
Contact point alignment	In the anterior sectors, the contact points are located near the incisal third and their sequence defines a curvilinear pattern.	YES
Color	Color is one of the cardinal elements of dental aesthetic recovery. It must always be evaluated in a much broader context, involving many other periodontal, labial, and skin parameters of the patient.	YES
Gingival scalloping	Gingival parabolas are decisive for the aesthetic effect of the frontal group. Orthodontic treatment can contribute to their harmonization.	YES
Negative space	A restrained smile enhances the characteristics of the teeth, while an excessive smile imparts an unattractive sense of emptiness.	YES
Arch geometry	There are several types of arch form related to individual craniofacial conformation that must be recreated or respected by orthodontic treatment.	YES
Buccal corridors o black tunnels	A restrained smile enhances the characteristics of the teeth, while an excessive smile imparts an unattractive sense of emptiness.	YES
Fibonacci golden proportion	It evaluates proportions by relating harmony to numerical values. In the dental field, it can find application in the evaluation of various dental and facial morphological parameters.	YES

## Data Availability

Not applicable.
